# ‘I’d have to fight for my life there’: a multicentre qualitative interview study of how socioeconomic background influences medical school choice

**DOI:** 10.1080/10872981.2022.2118121

**Published:** 2022-09-01

**Authors:** Eliot L Rees, Karen Mattick, David Harrison, Antonia Rich, Katherine Woolf

**Affiliations:** a Research Department of Medical Education, University College London, London, UK; b School of Medicine, Keele University, Newcastle-under-Lyme, UK; c Department of Health & Community Sciences, University of Exeter, Exeter, UK

**Keywords:** Selection, widening participation, choice, medical education, admissions

## Abstract

Students from lower socio-economic backgrounds who were educated in state funded schools are underrepresented in medicine in the UK. Widening access to medical students from these backgrounds has become a key political and research priority. It is known that medical schools vary in the number of applicants attracted and accepted from non-traditional backgrounds but the reasons for this are poorly understood. This study aims to explore what applicants value when choosing medical schools to apply to and how this relates to their socioeconomic background. We conducted a multicentre qualitative interview study, purposively sampling applicants and recent entrants based on socioeconomic background, stage of application and medical school of application. We recruited participants from eight UK medical schools. Participants attended semi-structured interviews. We performed a framework analysis, identifying codes inductively from the data. Sixty-six individuals participated: 35 applicants and 31 first year medical students. Seven main themes were identified; course style, proximity to home, prestige, medical school culture, geographical area, university resources, and fitting in. These were prioritised differently depending on participants’ background. Participants from lower socioeconomic backgrounds described proximity to home as a higher priority. This was typically as they intended to be living at home for at least part of the course. Those from higher socioeconomic backgrounds were more concerned with the perceived prestige of medical schools. Since medicine is a highly selective course, only offered at a minority of UK higher education institutions, these differences in priorities may help explain observed differential patterns of medical school applications and success rates by applicant social background.

## Introduction

Widening participation in medicine has become a priority in many countries over recent years. The aims of widening participation are to ensure the full talent pool of applicants is utilised, produce a diverse and representative workforce, improve social mobility, redress inequalities, promote social mobility, and ensure social justice[1]. The specific goals of widening participation vary by national context, depending on which groups experience inequality of access [1]. Countries such as Canada and Australia focus on increasing participation amongst indigenous peoples. In the UK, where data suggests the application and admission rates of ethnic minority applicants are disproportionately high [2], the greatest focus is on social mobility through widening participation by socioeconomic background [3]. Data from 2009–11 demonstrated that 80% of UK applicants to medical school were from students at 20% of UK high schools, with 50% of high schools having not sent any applicants to medical school in this period[4]. Furthermore, an analysis of UK-domiciled applicants in the same period demonstrated that there were three times as many applications from students in the most affluent quintile according to the index of multiple deprivation (IMD – a composite measure of deprivation in geographical areas in the UK [5]) compared to those in the most deprived quintile (36.4% and 12%, respectively) [6]. A quarter of applicants were from fee paying schools, whereas only 6% of school children are educated in the private sector [6,7] (see context section for definitions of school type). Over half of the successful applicants in these three admissions cycles were from the most affluent IMD quintile[6].

Differences between medical schools in the proportion of non-traditional applicants and entrants is rarely explored in the literature; however a retrospective analysis of application data for 2009/10 to 2011/12 identified an almost fourfold variation in the proportion of applicants and entrants whose parents were in the lowest two of five occupational categories as measured by the National Statistics Socio-Economic Classification (NS-SEC 4; lower supervisory and technical occupations, and NS-SEC 5 semi-routine and routine occupations) amongst the 22 medical schools they studied [6]. This raises questions about how applicants from different backgrounds are choosing which medical schools to apply to, and whether this impacts on their chances of being offered a place.

Most admissions research within medical education focuses on the psychometric properties of admission procedures, with relatively little attention given to applicant perspectives [8]. Within higher education it is recognised that more informed choices have the potential to increase students’ success within their studies, and can assist with social mobility [9]. Indeed, in the UK, supporting applicant choice of higher education has become a national imperative [9]. In selection to medicine, where competition is fierce, choice is likely to be even more important in influencing success. Here the potential consequences reach beyond under-match (in which applicants from the lowest socioeconomic groups are less likely than their more privileged counterparts with the same grades to apply to the most competitive universities [10]) to a significant risk of no offers at all. It is therefore critical that applicants are able to make well-informed choices [11], yet applicant choice of medical school has been the subject of little enquiry. Given the challenges faced in widening participation to medicine, and the likely importance of choice in success, understanding the choices of applicants from different backgrounds is of interest and importance.

One of the few studies that has looked at medical applicant choice in the UK was a qualitative study of applicants to medical schools in 2003. The authors identified three major medical school factors influencing applicant choice: academic features, location, and intangibles [12]. Applicant factors such as socioeconomic and educational background, were not considered in this study, yet it is likely that they are important. Studies from the broader higher education context indicate the importance of prestige and geography in applicants’ choice, and highlight differences in priorities amongst students from lower socio-economic backgrounds [11,13,14].

This study aims to explore what applicants value when choosing medical schools and how this relates to their socioeconomic background. Answering this question is the necessary first step in understanding how they go on to make choice decisions.

## Methods

### Design

We conducted a multicentre qualitative interview study of applicants and recent entrants sampled from eight UK medical schools, adopting a subjectivist inductive approach. Participants were invited to attend individual or group interviews according to their preference. After one group interview, we found that the depth of data was not comparable to the individual interviews, and so we prioritised individual interviews going forward. Data were analysed through framework analysis as described by Ritchie [15]. This paper is reported in accordance with the Standards for Reporting Qualitative Research [16].

### Context

In the UK, there are many different types of school that provide secondary level education (high schools) [17]. The most pertinent differences between these schools for the purposes of understanding medical school applications are how the schools are funded and who is eligible to attend. State schools are funded through national taxation. State funded, non-selective, schools (e.g., comprehensive schools) accept any children within a specified geographical area. State funded, selective, schools (grammar schools) accept students based on academic ability as measured on a national exam at age 11. Private schools (also known as independent schools) are funded through tuition fees and generally accept students based on academic ability. The majority of applicants to medicine apply directly after secondary school. Applicants can apply to up to four universities through a national application system(UCAS). There are currently 40 UK medical schools approved to issue primary medical qualifications. Applicants are typically shortlisted based on their secondary school academic attainment and performance on aptitude tests (most medical schools use the University Clinical Aptitude Test, with a minority using the BioMedical Admissions Test; the Graduate Medical School Admissions Test is used by medical schools offering a fast-track medical degree for students who have already completed an undergraduate degree in a different subject). Shortlisted applicants are invited to interview, typically either a panel interview or multiple mini interviews.

### Sampling and recruitment

We employed stratified purposive sampling [15]. We stratified by three characteristics that we considered pertinent to our research question: participants’ social backgrounds, their stage of application, and the medical schools they chose (or are choosing) to apply to (see below).

- **Social background**: We argue that ‘traditional’ and ‘non-traditional’ backgrounds are not binary phenomena, rather that applicants can be more or less traditional. Consequently, we have used a composite definition of ‘traditional’ as being applicants who attended fee-paying schools, whose parent(s) had higher education degrees (including medical degrees), and who were from areas of high higher education participation and/or from affluent areas. We have defined ‘Non-traditional background’ as applicants who attended state-funded secondary schools, who were First in Family to attend higher education, and who were from areas of low higher education participation and/or areas of high deprivation. These factors were chosen as they are criteria frequently used to confer eligibility for contextual admissions to medical schools in the UK [1,18]. Participants were categorised on an ordinal scale from non-traditional to traditional depending on how many traditional attributes they possessed.
- **Stage of application**: We aimed to recruit applicants from various stages of application ranging from those seriously considering applying to medical schools, to those that are currently studying medicine and were reflecting back on the application process. To ensure we captured the views of successful applicants, our sample also included current first year medical students with recent experiences of applying to medical school.
- **Medical school(s) applied to**: Previous research has demonstrated that applications to UK medical schools in 2013 exhibited systematic groupings, with seven clusters of medical schools identified[19]. In order to capture perspectives from participants who had or were likely to apply to all UK medical schools, one school from each of the seven clusters was selected for recruitment for the current study. Due to difficulty with recruitment at one medical school (resulting from limited access to potential participants) a second school from that cluster was selected, hence there are 8 medical schools in total.

The method of recruitment varied between medical schools, and between groups within medical schools and included mass emails, attendance at open days, attendance at offer holder visit days, attendance at widening participation schemes, and shout outs in lectures. Members of the research team abstained from admissions activities during the data collection period and emphasised that participants’ contributions would have no bearing on admissions decisions.

### Data collection

We conducted individual semi-structured interviews and a single group interview between January 2018 and January 2019. Interviews were conducted face-to-face or via telephone, according to participant preference. The interviews were predominantly conducted by the first author, with other members of the research team (KW, AR, HS, LK) conducting a small quantity when he was not available. The interviews followed a topic guide (supplemental appendix) tailored to whether the participant was an applicant or recent entrant to medical school. We conducted three pilot interviews with previous medical students after which minor refinements were made to the topic guides. The research team members contributing to interviews met to discuss and practice the questions in the topic guide. The interviews were audio recorded, transcribed verbatim, pseudo-anonymised, and identifiable information was redacted.

A background survey was completed at the start of the interview to identify indicators of social background at an individual level (e.g., parental higher education experience), area level (e.g., locality deprivation derived from participant postcode), and school-level (e.g., state or private schooled). These were used to categorise participants’ social backgrounds as more or less traditional, as described above.

### Analysis

We analysed the transcripts using framework analysis [15], the core features of which are that analysis remains grounded in the data, the process allows comprehensive coverage of the data, and facilitates within and between case comparisons. Framework analysis was used as it makes it possible to analyse large qualitative datasets while balancing the breadth of all participants and the depth of individual accounts. We followed the five stages of data management in framework analysis described by Ritchie *et al*. [15].

- *Familiarisation*. One author (ER) read all the transcripts and listened to the recordings to check accuracy of transcription. A second researcher (KW) read through all the transcripts.
- *Constructing an initial thematic framework*. Four researchers (ER, KM, DH, KW) each independently analysed four transcripts to identify thematic codes. We met to discuss themes and generate an initial thematic framework. We then uploaded all transcripts to NVivo (NVivo qualitative data analysis Software; QSR International Pty Ltd. Version 10, 2012) to facilitate analysis. We then independently coded a further two transcripts to evaluate how well the initial thematic framework was working, discussed, and refined the initial framework.
- *Indexing and sorting*. One researcher (ER) then coded all the transcripts to the thematic framework, and a second researcher (KW) coded 20%.
- *Reviewing data extracts*. Data extracts for each code were reviewed for coherence and the thematic framework was finalised. This included the merging of similar themes and the splicing of those with disparate data.
- *Data summary and display*. We then created a framework matrix of themes and subthemes against individual participants grouped by socioeconomic backgrounds. One researcher (ER) created a precis (e.g., ‘wanted to be near the coast’) for each participant’s comments on each subtheme. In order to account for patterns within our data we reviewed each participant’s comments about each theme and attributed them to one of the following categories: not discussed, discussed but not described as a priority, described as a significant consideration, described as their top priority. We inferred the absence of discussion regarding a theme as indicating that it was low priority for the participant.

Following these steps, we turned to abstraction and interpretation. This involved describing the findings from each theme, and using both the data summaries and the raw data within each theme to identify linkage and account for patterns.

### Reflexivity

Throughout our analysis we were sensitive to the potential for our backgrounds to influence the findings we drew. Early within the research process we met to discuss our previous experiences and orientation to the research study. We completed a tool consisting of orienting questions aimed at optimising reflexivity in team based qualitative research [20]. Furthermore, the research team’s characteristics are reported here. ER is a medical doctor, PhD candidate, and lecturer. He attended a state-funded school and was unsuccessful in his first attempt at applying to medical school. KM is a medical education researcher with a previous background as a clinical scientist who attended a state-funded secondary school. DH is an education researcher with a background as a secondary school teacher who attended a state-funded secondary school. AR is a medical education researcher, with a background in health psychology who attended a state-funded secondary school. KW is a medical education researcher with a background in psychology who attended a state-funded sixth form after an international secondary school.

### Ethics

This study was approved by the UCL Research Ethics Committee reference: 0511/013 and received approval via chair’s action at the other seven medical schools. All participants gave informed consent to participate.

## Results

### Participants

Sixty-six individuals participated in 61 individual interviews and one group interview. Thirty-five participants were applicants, and 31 were current first year medical students. The interviews lasted a mean of 54 minutes (range 22–113). Details on the participants are presented in Table 1.

**Table 1.** Details of participants.

	Non-traditional (*No traditional attributesa)*	Less traditional *(One traditional attributea)*	More traditional *(Two traditional attributesa)*	Traditional *(Three traditional attributesa)*
Applicant	7	13	15	0
Recent entrant	3	9	17	2

*a Traditional attributes defined as: i)attended fee-paying schools ii) parent(s) had higher education degrees, iii) From areas of high higher education participation and/or from affluent areas*

### Overview of themes

We constructed seven main themes (Table 2). Across participants, **course style** was often described as the top priority or at least a significant factor in choice, with **proximity to home, prestige**, and **geographical area** also important to many. **Medical school culture** and **university resources** were discussed by most participants, although the latter was only rarely considered a priority.

**Table 2.** Themes identified listed in order of priority across all socioeconomic groups.

Theme	Definition	Sub themes	Socioeconomic group differences
Course style	Course/curricular style offered by a medical school.	Problem based learning Early clinical experience Cadaveric dissection Cohort size Extra degrees	Prioritised highly by all participant groups.
Proximity to home	How close a medical school was from participants’ home.	Minimising costs Family support University experience	Prioritised higher by non-traditional and less traditional participants.
Prestige	How prestigious a medical school was perceived to be.	Defining prestige Importance of prestige	Prioritised higher by traditional and more traditional participants
Medical school culture	Perceived culture and feel of a medical school.	Values Feel Satisfaction Support	Prioritised higher by non-traditional and less traditional participants.
Geographical area	The area in which a medical school was situated, including opportunities for extracurricular activities.	City Things to do Living expenses Familiarity Safety	Prioritised higher by traditional and more traditional participants
University resources	Campus and facilities of a medical school, including cost of accommodation.	Facilities Campus Accommodation	Lower priority for all participant groups
Fitting in	Whether participants felt they would fit in at a medical school.	Socially Class Ethnicity	Prioritised higher by non-traditional and less traditional participants.

Clear groupings by participant social background could be seen for some themes. **Proximity to home** was more often a top priority for non-traditional and less traditional participants, whereas **prestige** tended to be particularly important for traditional and more traditional participants. Geographical area was also given relatively more weighting by more traditional participants. **Fitting in**, while often not discussed and never described as a top priority, was an important consideration for some participants in the less traditional group.

Each theme will now be presented in order of the extent to which they were described as priorities across the whole sample. In each section, the theme will first be described, followed by one or more illustrative quotes.

### Course style

Participants across all groups often expressed preferences for a course or curriculum style; some described ruling out medical schools based on their use or not of Problem-Based Learning (PBL). In this sample participants described their perceptions of medical schools either offering ‘PBL’ or ‘traditional’ curricula and these descriptions were used throughout the interviews. While this may not reflect how course leaders would distinguish curricula these terms are used here to portray participants’ perceptions most accurately. PBL was described as more popular than traditional curricula, with participants wanting small group work and the opportunity to develop independent learning skills, and to avoid lectures. A preference for traditional courses was largely based on scepticism about PBL rather than a desire for lectures. Despite this, participants expressed concerns regarding PBL about making mistakes, not knowing how much depth to learn, lack of teaching, and peers jeopardising their learning with errors. Early clinical experience was described by the vast majority as expected to be enjoyable, perceived to be putting science in context, and aiding the development of clinical and professional skills. Although several participants described wanting a traditional course with early clinical experience, the few who did not want early clinical experience were all negative about PBL, and wary of feeling underprepared for clinical experience without basic science knowledge.

Despite its stated importance, it is uncertain how much course/curriculum style determined medical school choice. Many participants reported applying or having applied for medical schools with a different style from the one they said they wanted, or the four medical schools they had chosen had different styles. It may be that participants were stating what they believed others valued, perhaps because they did not know what a particular style would entail before experiencing it. For example, Nicole (less traditional medical student) based her beliefs about PBL on ‘stories’ from current students and Joseph (more traditional medical student) could not explain why he had wanted a school that did dissection:

I think the main thing that put me off was the stories people told me about their learning style in classes, because for a while they were doing 100% - I think it’s called Problem Based Learning. Someone described it to me as paying £9,000 a year to look things up on Wikipedia […] So that kind of put me off straight away

(Nicole, less traditional medical student)

Joseph: So the reason I was attracted to this medical school is they have full body dissection, so they have a dedicated anatomy facility that helps you to learn the body in its normality.

Interviewer: And why is that important?

Joseph: To be honest I don’t know actually. A lot of people, because you don’t really know until you’ve been in something how it actually works, and nobody goes to two medical schools because you can’t go to two medical schools.

(Joseph, more traditional medical student)

### Proximity to home

Almost all participants discussed the proximity of medical school to home as being important to choice. Most preferred a school close to home to minimise costs and to receive and/or provide family support. Several, all from non-traditional or less traditional groups, only considered the four nearest schools so they could live at home; none discussed how this might affect their chances of getting an offer. Several participants said moving away from home was important to develop independence as an adult and have a ‘university experience’, although even those from more traditional backgrounds considered the financial costs of this.

A few participants discussed feeling social pressure to move away. For example, Jack (less traditional medical student) described how he had wanted to stay close to home so that he could live with his parents to avoid the rented accommodation costs. Pressure from teachers and peers to apply to more distant schools, and being the first in his family to study at university made him fear missing out on ‘*this uni experience that everyone goes on about and that my family hadn’t really had*’. He successfully applied to his nearest school (and more distant schools) but felt he had compromised by living in halls for the first year for the experience. His parents rented out his bedroom and Jack got a part time bar job to pay the accommodation fees. He described this as a real challenge when trying to balance the academic demands of the course. He subsequently left his job and planned to return to his parental home for the rest of the course.

### Prestige

The prestige of a medical school was important to most participants, especially those from more traditional and traditional backgrounds. Some described prestige as being important at the start of their application when they had little else to consider, but other priorities emerged as they learned more about different medical schools. Participants who prioritised prestige felt it would improve their job prospects and give them a better learning experience. They also reported wanting to feel proud of themselves and impress their peers. Others, often from less traditional backgrounds, did not feel that a school’s prestige would affect their future career prospects as a doctor, and believed prestige was unrelated to teaching quality.

Participants inferred prestige from whether they had heard of a medical school (or the university/teaching hospital to which it was attached); how new (or old) it was; and how others (teachers, parents, and doctors they talked to) described the course and the performance of its graduates. Membership of the research-intensive Russell Group of Universities [21] was also a quick way to identify whether a university was prestigious or not, as described by Megan (traditional medical student) whose doctor parents ‘crossed off all the non-Russell Group [medical schools]’. Many participants used university rankings to infer prestige, although some felt this was socially undesirable, and others were uncertain how valid such rankings were.

### Medical school culture

The ethos and values of a school, the student support offered, the cohort size and general ‘feel’ of different schools were significant considerations for most. Participants tended to establish a school’s culture via experiences with staff and students during medical school visits, although participants also used satisfaction rankings.

Many participants felt tension between wanting prestige but also wanting a nurturing and supportive environment that did not value academic competitiveness above all else. This was particularly prevalent amongst participants from non and less traditional backgrounds, such as James and Andrei (both less traditional):

A perfect medical school was one that was going to […] a supportive learning environment that was conducive to learning. […] I’m going to be doing a very, very difficult degree, and I want to make sure that my academic staff are looking after me, rather than on my back.

(James, less traditional medical student)

I felt like I’d have to fight for my life there [at university X], just elbows and kicking […]. It’s sort of more highly regarded, I think, so I’d kind of built up a mindset that I’d have to struggle to kind of amount to anything. […] [At university Y] everyone else seemed more friendly and more approachable, so I think that was a big factor in it.

(Andrei, less traditional medical student)

### Geographical area

The geographical area of a medical school was important for most participants, who felt it would impact on their experience academically and socially. Although some participants wanted to live somewhere they were familiar with, many, especially those from more traditional backgrounds, wanted to move somewhere new for a ‘university experience’, as discussed above.

Cost of living, particularly in cities with their varied extra-curricular opportunities, was a consideration for many, although participants from non-traditional and less traditional backgrounds appeared more prepared to forego extra-curricular activities to minimise costs. Omar (less traditional medical student) describes a thought process shared by several participants who recognised London students were eligible for a larger student loan to assist with the higher cost of living, but were put off by the prospect of accumulating more debt:

My mum was saying, why are you bothering to go to [London university] when you could just get a different university where it’s cheaper? […] She was like, ‘oh you’re just going to be in more debt’. Because obviously, for accommodation you take a higher loan. Obviously other things are more expensive [in London].

(Omar, less traditional medical student)

### University resources

Most participants discussed university campus and buildings, medical school facilities, and student accommodation, and many were impressed by new buildings, although this was not a top priority. Student accommodation was discussed in terms of cost, perceived quality, and distance from teaching buildings. A few participants from non and less traditional backgrounds decided against particular schools based on the cost of the student accommodation although they did not discuss the cost of accommodation after the first year when students typically move off campus.

[University accommodation] were all either self-catered or too far from the campus, so they were either expensive or too far.

(Caitlin, less traditional applicant)

### Fitting in

The majority of participants discussed wanting to fit in academically and socially at medical school, although this was rarely a first priority. Participants described wanting to go to a medical school where there were people like them, or to a place that valued difference. Several participants from non- and less-traditional backgrounds described wanting to go to a medical school with students from similar socioeconomic backgrounds; some participants from state-funded schools were wary of medical schools where they thought the majority would have been privately educated.

But I’ve got a friend who got into [University] in London, he’s been to the exact same nursery, primary, and secondary and then college with me. And out of all his flat, I think he’s the only one who went to a state comprehensive. And I think they call him ‘Compy’ or something like that.

(Jack, less traditional medical student)

Several participants from ethnic minority backgrounds described concerns that they wouldn’t fit in at certain schools due to a lack of diversity, and a few specifically discussed wanting to attend an ethnically diverse medical school.

Perceptions of diversity were generally based on marketing materials and experiences on open days, visit days, and work experience at teaching hospitals associated with medical schools. Maria (more traditional student) and Francesca (less traditional applicant) told of how they had found themselves to be the only black people at open days or on work experience:

Just for the open day in general, when I got there, there were no other black people who were in the congregation. And they had two open days but on that day, I would say there were at least 150 people. Although there were other people from ethnic minorities, I just questioned if I would feel a sense of belonging there.

(Maria, more traditional medical student)

There were just no black females. Barely any people who were black, which was just like … It would be a bit hard, I feel like I wouldn’t fit in.

(Francesca, less traditional applicant)

We identified linkage between the seven themes that provide two broader categories of factors. Firstly, those that relate predominantly to the medical school and the course experience (course style, prestige, medical school culture, and fitting in). For example research-intensive universities with traditional courses were often perceived as more prestigious, and prestigious schools were felt to value high grades and competition over friendliness and supportiveness, and could be perceived as having more students from private schools and less ethnic diversity.

Secondly, those that relate more to the location in which the medical school is situated and the implications of this. For example, proximity was very important to participants who wanted to live at home, often for financial reasons, and this was prioritised over the extra-curricular or social activities offered by medical schools in costly urban areas. Those living at home for financial reasons could also be put off a medical school by the cost of student accommodation.

## Discussion

### Summary of results

In this large qualitative study of medical applicants and first year medical students across the UK, we aimed to explore what applicants value when choosing medical schools and how this relates to their socioeconomic background. We found that in choosing which medical schools to apply to participants valued seven main factors: course style, proximity to home, prestige, medical school culture, geographical area, university resources, and fitting in. These were prioritised differently depending on participants’ social background. Proximity to home seemed to be more of a priority to those from less traditional backgrounds, who were more likely to want to live at home, at least partly to minimise costs. They reported being less likely to prioritise a medical school based on its extra-curricular or social opportunities and tending to value medical schools they perceived as friendly and supportive rather than those they perceived as competitive and valuing academic prestige above all else. By contrast, prestige was cited as a higher priority for those from more traditional backgrounds, who were more likely to want to move away from home and study somewhere they thought would provide an enjoyable university experience.

Participants frequently stated that a particular course or curriculum style strongly influenced their choice, but it was uncertain how much this actually determined choice among participants, who had limited experience of different pedagogical styles. Fitting in socially at university was more important to participants from state schools and ethnic minority groups, although it was typically not a top priority for any participants. Concerns about perceived fit and belonging are important for minority groups in higher education, including ethnic minorities but also people from other underrepresented groups. Differences regarding ethnicity ‘fit’ are likely to be nuanced and play out differently in the UK to other contexts.

We have identified factors important in medical school choice; however, several participants recognised that the *‘*perfect’ medical school for them might not exist, or if it did, it might also be desirable to other applicants and be more competitive to get in to. The ways in which applicants adjust their priorities as they choose which four medical schools to apply to is likely to depend on the amount of resources and constraints (financial, academic, social and personal) they feel they have. For example, in this study we saw how some less-traditional applicants were concerned that they would struggle to manage academically at a prestigious medical school, and therefore prioritised medical schools they perceived to be supportive and friendly over highly prestigious medical schools they felt valued academic competitiveness above all else. By contrast, it is likely that applicants who consider themselves to have very high grades may feel they are able to prioritise prestige more than an applicant who feels their grades are lower. Similarly, applicants with more financial support may feel able to move away from home to study medicine, whereas applicants with caring responsibilities may feel unable to leave home. What applicants from different backgrounds consider their resources and constraints to be, and how these perceptions influence their priorities and medical school choices will be explored in a subsequent paper from the same programme of research.

### Comparison with previous literature

There have been relatively few studies describing applicants’ priorities in choosing medical schools. Two previous multi-institutional studies of UK medical school choice are McManus et al’s [22] questionnaire study of applicants in 1990 and Brown’s qualitative interview study of applicants in 2003 [12]. Both studies identified medical school reputation (academic and non-academic) and location (proximity to home and the desirability of a particular location) as key factors in choice, which reflects our findings. McManus’s study was conducted before the widespread introduction of problem-based learning into UK medical education, however Brown’s study found, as we did, that course structure/style/curriculum was stated as a factor in choice. Brown’s third theme, ‘intangibles’ included: the ‘feel of the medical school’, which is similar to our **Medical school culture** theme; ‘personal compatibility’, which is similar to our **Fitting In** theme; and ‘personal recommendation’, which is covered within our **Prestige** theme. Two smaller single-institution studies with medical students similarly reported course style, geographical area, friendliness and social life as key reasons for choice [23,24]. A study from the USA reported that medical school reputation was a key factor in choice [25]. In the Netherlands, where there are two main distinct types of medical school selection procedures, Wouters and colleagues found that the selection procedure used was a major factor in choice [26].

In general there has been little exploration in the literature of how priorities differ by social background amongst medical applicants; however, Wouters et al found first generation students are less likely to choose medical schools based on the curricula they offer compared to those with familial experience of higher education [26], which differed from our finding that the emphasis placed on course style did not differ by social background. In the USA, applicants from ethnic minorities reported the diversity of the student body and the diversity of the faculty as more positive reasons for choosing medical schools [25]. This is similar to our finding that applicants from less traditional backgrounds and from ethnic minority groups were more concerned with wanting to fit in at medical school.

Research in the wider discipline of higher education has identified that applicants from lower socioeconomic backgrounds make substantially different choices [11], most significantly they apply to and attend less prestigious universities given their grades [10]. One explanation may be that applicants from lower socioeconomic backgrounds have little choice but to prioritise other features for financial reasons, notably proximity to home [27]. Differences in other forms of capital, for example social capital [28], are likely to also play a significant role.

### Strengths and limitations

This large qualitative study includes participants from around the UK who were applying to or were studying at eight UK medical schools. These medical schools varied in prestige, curriculum style, cohort size, geographical location, and entry requirements. This provided a diversity of views absent from single-site studies of current students, and the qualitative approach allowed us to explore the complexities of choice within a socially and geographically diverse sample of applicants and students. Few studies have looked at applicant perspectives regarding medical school choice prior to admission to medical school, by doing so we were able to include a more diverse sample including those who may be unsuccessful in their applications. Although qualitative research does not require a sample to be representative of the wider population, the data collected and conclusions drawn inevitably reflect the participants interviewed. Therefore the makeup of the sample is important to reflect upon, in case certain important perspectives have been missed. Our study had a relatively high proportion of participants from non-traditional and less-traditional backgrounds, who are typically under-represented at medical school, which enabled us to explore how priorities differed across social groups. More participants categorised as ‘traditional’ might have enabled us to better explore perceptions amongst this group. We suspect we may have had fewer of these participants partly due to our recruitment strategy and partly due to a perceived stigma of being ‘traditional’ in the current culture of widening access. It is likely that the views of current students in our sample were affected by their experiences of being accepted or rejected by particular medical schools; however a further strength of our study is that it also allowed us to explore perceptions prospectively among participants who had not yet applied to medical school, as well as those who did not know the outcome of their application, which is rare in the literature.

### Implications for policy, practice and future research

If more traditional applicants prioritise prestige and social/extra-curricular opportunities, whereas less traditional applicants prioritise proximity to home and supportiveness, this is likely to result in different application patterns by social background, and potentially in different success rates, as seen in Steven et al’s (2016) analysis of administrative data [6]. This is potentially a particularly important issue for medicine compared to other degrees because only a minority of higher education providers in the UK offer medical degrees (currently 40 out of 271) [29], which means applicants may not have four medical schools within commuting distance from home. Medicine is also extremely competitive, with only a minority of applicants obtaining one or more offers and becoming a doctor. Increasingly applicants need to use their four choices strategically to maximise their chances of getting in.

Current widening participation initiatives include encouraging non-traditional applicants to consider medicine as a subject [30], supporting applicants preparing for admissions tests, reducing grade requirements for applicants from disadvantaged social backgrounds (‘contextual admissions’), and providing financial support via scholarships [31]. Our findings suggest that there are a number of additional activities medical schools could do which might help applicants from less traditional backgrounds be successful.

Medical schools could provide more relevant and accessible information about the aspects of many different medical schools/courses that are likely to be important to non-traditional applicants, as well as helping them consider the potential implications of prioritising some factors over others. Medical schools could tailor aspects of their provision still further to make it more attractive for students from non-traditional backgrounds to apply and easier for them to accept offers. No applicant should be concerned that they might face difficulties at medical school on the basis of their socioeconomic background, ethnicity, or any other personal characteristic. A study of photos on UK medical school websites identified that students from ethnic minorities were underrepresented compared to the UK population [32] which is likely to perpetuate the feeling that they may not fit in and may be indicative of the culture of that medical school. Medical schools that do have diverse student populations should emphasise this within their promotional materials.

As alluded to earlier, variations in different forms of capital are likely to influence choice decisions. Further research is required to better understand this. Similarly, further research explicitly exploring how applicants from different ethnicities evaluate and prioritise fit when making choices in applications is needed. This will be particularly important as admissions criteria change in attempts to widen access to medicine.

## Conclusions

Since medicine is a highly selective course only offered at a minority of UK higher education institutions, differences in the priorities of medical applicants from different social backgrounds may help explain observed differential patterns of medical school applications and success rates. To ensure students do not feel precluded from studying at certain medical schools because of their social background and have an equitable opportunity to study medicine, medical schools could provide information and guidance that is more relevant to applicants from non-traditional backgrounds and tailor their provision to be more inclusive. In this way medical schools can aim for a genuine meritocracy in medical school admissions, whereby applicants from all social backgrounds have the same ability to select medical schools to match their wants and needs.

## Supplementary Material

Supplemental Material
