# Supplementary material for: ‘I’d have to fight for my life there’: a multicentre qualitative interview study of how socioeconomic background influences medical school choice
Source: Med Educ Online. 2022 Sep 1;27(1):2118121. doi: 10.1080/10872981.2022.2118121 (PMC9448433; doi:10.1080/10872981.2022.2118121)
Supplement: Supplemental Material [file ZMEO_A_2118121_SM4325.docx]

**Interview questions one-to-one applicants**

Thank you for taking part in the UK Medical Applicant Cohort Study. The aim of this research is to find out how people from different backgrounds choose which medical schools to apply to.

I’d like to audio record this interview and take some notes to help me accurately remember what was said. The recording will be sent to a professional independent transcriber. We will anonymise the transcript, which means we will remove anything that might identify you. All notes will also be anonymised.

What you say will be kept confidential – we won’t share it with anyone outside of the research team. We will only publish it in a way that means that nobody can identify you.

Over the next half an hour or hour so, I’m going to be asking you a series of questions. There are no right or wrong answers, I just want to hear your opinions and experiences.

If at any time you want to stop, just let me know and we will stop.

[TURN ON TAPE]

Are you happy to go ahead?

**1. Tell me a bit about yourself**

1. Whereabouts in the country do you live? *(which city/town/village is that near?)*
2. Are you from there originally or did you grow up somewhere else?

(*If somewhere else):* Where did you grow up?

1. Who do you live with at the moment?

**2. Now I’m going to ask you about choosing to study medicine**

1. *(if not yet applied)* How certain are you that you will apply to study medicine?
2. What is it about studying medicine or being a doctor that appeals to you?
3. Is there anything about studying medicine or being a doctor that puts you off ?
4. If you get into medical school, do you have an idea of where you ultimately would like to get to career-wise? *(do you know what specialty you might like to work in? Whereabouts you might want to work?)*

**3. I’m going to ask some questions about choosing a medical school.**

1. Can you describe to me what the perfect or ideal medical school would look like for you?
   1. What things about it appeal to you?
2. Thinking about where you might actually apply/have actually applied, can you talk me through how you are considering/considered which medical schools to apply to?
3. Which medical schools do you think you might apply to/have you applied to?
   1. Which is your first choice?
4. Thinking about that first choice, what do you like about it?
5. Is anything about it off-putting?
6. What about the other medical schools you mentioned, what do you like about them? *(compare and contrast their choices e.g. if they mention location, ask which is best location-wise)*
7. Did anything put you off them? *(Compare and contrast their choices e.g. if they mention being hard to get into, ask which is hardest to get into)*
8. What was it about your first choice that makes you prefer it to the others?
9. Bearing all that in mind, when you are/were choosing medical schools to apply to, what are/were the most important considerations for you? Why?
10. Were there any medical schools you immediately ruled out? Why?
11. If you knew/had of known that you were guaranteed get a place at any medical school in the country, which one would you have chosen? Why?
    1. *(if they haven’t already mentioned it)* Why aren’t you applying/didn’t you apply there?
12. Is there anything you think might work in your favour and help you get a place at medical school? How important do you think that is?
13. Is there anything you think might work against you? How important do you think that is?
14. If you don’t study medicine, what do you think you will you do instead?

**4. I’m going to ask you some questions about the information you used or are using to choose a medical school and where you got that information.**

1. What information or resources have you used or will you use to help you choose which medical schools to apply to?
2. How did you know where to get that information or access those resources? (*for example, if it’s a website, who told them about it? If they went on a visit, how did they arrange that? If they spoke to someone, how do they know that person?* *If they’re part of a medical school widening participation programme, how did they hear about that programme?* *ask about each resource*)
3. Have you spoken to anyone else about which medical schools you might apply to?

(*If YES*): Who?

(*For each person mentioned*): (*if relevant)* How did you meet that person? How much did they influence your choices of medical school?

*(If NO)*: Are you planning on talking to anyone about choosing which medical schools to apply to?

*(If YES)*: Who?

*(for each person):* Why them? *(if relevant)* How will you get in touch with them?

(I*f NO)*: Why not?

1. Of all the information you’ve had so far, which was most useful in helping you decide which medical schools to apply to? Why?
2. If you were to advise someone else about getting information to help them choose which medical schools to apply for, what would you suggest?

**5. Thank you very much. That’s all my questions.**

1. Is there anything else you want to tell me about? Or any questions you have for me?

**Interview questions one-to-one medical students**

Thank you for taking part in the UK Medical Applicant Cohort Study. The aim of this research is to find out how people from different backgrounds choose which medical schools to apply to.

I’d like to audio record this interview and take some notes to help me accurately remember what was said. The recording will be sent to a professional independent transcriber. We will anonymise the transcript, which means we will remove anything that might identify you. All notes will also be anonymised.

What you say will be kept confidential – we won’t share it with anyone outside of the research team. We will only publish it in a way that means that nobody can identify you.

Over the next half an hour or hour so, I’m going to be asking you a series of questions. There are no right or wrong answers, I just want to hear your opinions and experiences.

If at any time you want to stop, just let me know and we will stop.

[TURN ON TAPE]

Are you happy to go ahead?

**1. Tell me a bit about yourself**

1. Can you tell me a bit about yourself?
2. When you’re at medical school, do you live at home or in halls or somewhere else?

*(if live at home)* Whereabouts in the country do you live? *(which city/town/village is that near?)*

(*If don’t live at home)* Whereabouts in the country do you live when you’re at home i.e. not at medical school? *(which city/town/village is that near?)*

1. Are you from there originally or did you grow up somewhere else?

*(If somewhere else):* Where did you grow up?

1. Did you live there or somewhere else when you were applying to medical school?

**2. Now I’m going to ask you about choosing to study medicine**

1. Can you tell me about how you came choose medicine? *(how old were you when you first considered it? Did you talk to anyone about it?)*
2. When you were applying to medical school, what was it about studying medicine or being a doctor that appealed?
3. At the time, was there anything about studying medicine or being a doctor that put you off?
4. When you were applying, did you have any idea of where you ultimately would like to get to career-wise? *(what specialty you wanted to work in?)*

**3. I’m going to ask some questions about how you chose which medical schools to apply to.**

1. Think back to when you were applying to medical schools, can you describe to me what the perfect medical school would have looked like for you?
   1. What things about it would have appealed to you?
2. Can you tell me how you came to decide which medical schools to apply to?
3. Was [*current medical school*] your first choice?
   1. *[if current med school not first choice]* Which was your first choice?
4. Thinking about your first choice of medical school, what did you like about it?
5. Can you tell me about anything that was off-putting?
6. What did you like about the other schools you applied to? *(Compare and contrast their choices e.g. if they mention location, ask which is best location-wise)*
7. Did anything put you off them? What? *(Compare and contrast their choices e.g. if they mention being hard to get into, ask which is hardest to get into)*
8. What was it about your first choice that made you prefer it to these other schools?
9. Bearing all that in mind, when you were choosing medical schools to apply to, what were the most important considerations for you?
10. Were there any medical schools you immediately ruled out? Why?
11. When you were applying, if you’d have known that you were guaranteed get a place at any medical school in the country, which one would you have chosen? Why?
    1. *(if they haven’t already mentioned it)* Why didn’t you apply there?
12. When you were applying, was there anything you thought might work in your favour and help you get a place? How important do you think that was?
13. Was there anything you thought might work against you? How important do you think that was?
14. What would you have done if you didn’t get in to any of these medical schools?

**4. I’m going to ask you some questions about the information you used to choose a medical school and where you got that information.**

1. As you know there are lots of medical schools, how did you find out what each of them was like?
2. When you were choosing medical schools, what information or resources did you use to help you make those choices?
3. How did you know where to get that information or access those resources? (*for example, if it’s a website, who told them about it? If they went on a visit, how did they arrange that? If they spoke to someone, how do they know that person?* *If they’re part of a medical school widening participation programme, how did they hear about that programme?* *ask about each resource*)
4. Did you speak to anyone / anyone else about which medical schools you might apply to?

*(If YES)*: Who?

*(for each person):* How much did what they have to say influence you?

*(If NO)*: Why not?

1. If you were to advise someone else about getting information to help them choose which medical schools to apply for, what would you suggest?

**5. Thank you very much. That’s all my questions.**

1. Is there anything else you want to tell me about? Or any questions you have for me?
